# Design and Analysis of Active Metamaterial Modulated by RF Power Level

**DOI:** 10.1038/s41598-020-65318-0

**Published:** 2020-05-26

**Authors:** Ratanak Phon, Sungjoon Lim

**Affiliations:** School of Electrical and Electronic Engineering, Chung-Ang University, Heukseok-dong, Dongjak-gu 156-756 Republic of Korea

**Keywords:** Aerospace engineering, Electrical and electronic engineering

## Abstract

In this paper, a radio frequency (RF)-power-modulated active metamaterial loaded with a nonlinear Schottky diode is presented. Its operating mode is a function of the incident power level. It is switched by a change in the operating state (i.e., on/off) of the Schottky diode, which is directly triggered by a change in the incident power level. For instance, when a low-power RF radiation is incident on the proposed metamaterial, the Schottky diode is turned off, and the metamaterial passes a 2 GHz signal in the pass-band mode. By contrast, when a high RF power is incident, the diode is turned on, and the metamaterial reflects all frequencies in the reflection mode. The proposed active metamaterial was analysed by performing numerical simulations for both low- and high-power modes, and the proposed concept was successfully demonstrated by circuit analysis, full-wave simulation, and experimental results.

## Introduction

Metamaterials, also known as artificial electromagnetic (EM) structures, have received considerable attention owing to their unusual properties, which are not found in nature. For instance, the EM parameters permittivity and permeability of a metamaterial can be artificially tailored to be negative, which would render the metamaterial suitable for the fabrication for realizing a super lens and for invisibility cloaking^1^. Electrically small antennas and beam-steering leaky-wave antennas based on metamaterials have been proposed^2^. A perfect electromagnetic absorber can be realized by manipulating the impedance of a metamaterial to match the impedance of free space^3^. Metamaterial absorbers can be used to reduce the radar cross-section (RCS) in stealth technology^4–6^, increase the performance of photodetectors^7,8^, and enhance light absorption by solar photovoltaics^9,10^ and thermophotovoltaics^11,12^.

Active reconfigurable metamaterials are useful since they are multifunctional without additional fabrication. Various tuning and switching techniques have been used to change their frequency, polarization, propagation direction, and other characteristics. For example, spring resonators and motors have been used to control or actuate the structure of a system to tune the bandpass and bandstop responses of the metamaterials; control or actuation was achieved by varying the length of the spring resonators and the speed of the motors^13,14^. Microfluidic technology has been proposed to introduce tuning or switching characteristics of the metamaterials. Through the control of the movement of liquid metal droplets in tubes, frequency and phase tuning can be continuously achieved^15^. Furthermore, switching between four different working states (dual-polarized all pass, single-polarized low pass, single-polarized bandpass, and dual-polarized bandpass) is possible by injecting liquid metal into the top and bottom microchannels of a metasurface^16^. However, the reconfiguration speed of the above techniques is not insufficient. Recently, a graphene-based electrically reconfigurable metamaterial was presented with high potential for use in future technologies for controlling the behaviour of a structure, especially in the terahertz region. For example, a frequency tuned absorber was obtained by controlling the surface resistance of the graphene^17^. This technology is useful in the high-frequency regime, while the other aforementioned technologies are difficult to realize. Rather than frequency tuning, the polarization rotation angle of a bandpass graphene frequency-selective surface can be tuned at terahertz frequencies by changing its chemical potential^18^. The reader is referred to References ^19–21^ for examples of metasurfaces based on other active materials. However, many researchers find the conventional technique of using active components (varactors or p-i-n diodes) to be more reliable and easier to use for realizing metasurfaces with multifunctional characteristics^22,23^ since the operating state can be easily tuned/switched by controlling the external bias. For instance, a previous study proposed a multifunctional metasurface capable of three different functions, namely, transmission, reflection, and absorption. The functions could be controlled by using the bias voltage of diodes on the top and bottom layers of the metasurface independently^24^. In another study, two different types of diodes (varactors and p-i-n diodes) located on the top and bottom layers were separately used to achieve switching and tuning between the transmission and absorption modes^25^. The above-mentioned design can be implemented to achieve self-reconfigurable or power dependent characteristics by designing with additional sensing circuits^26,27^. However, these metasurfaces require a biasing configuration and an external power source to control their operating state, rendering them costly and difficult to design. Recently, nonlinear devices were reported where their EM functionalities depending on the incoming power levels^28–32^. For example, power-dependent metasurface that absorbed high-power but not low-power surface waves^29,30^. On another hand, a metamaterial-based nonlinear multiconductor transmission line is reported that achieves broadband switchable between transmission and reflection modes^31,32^.

**Figure 1:**
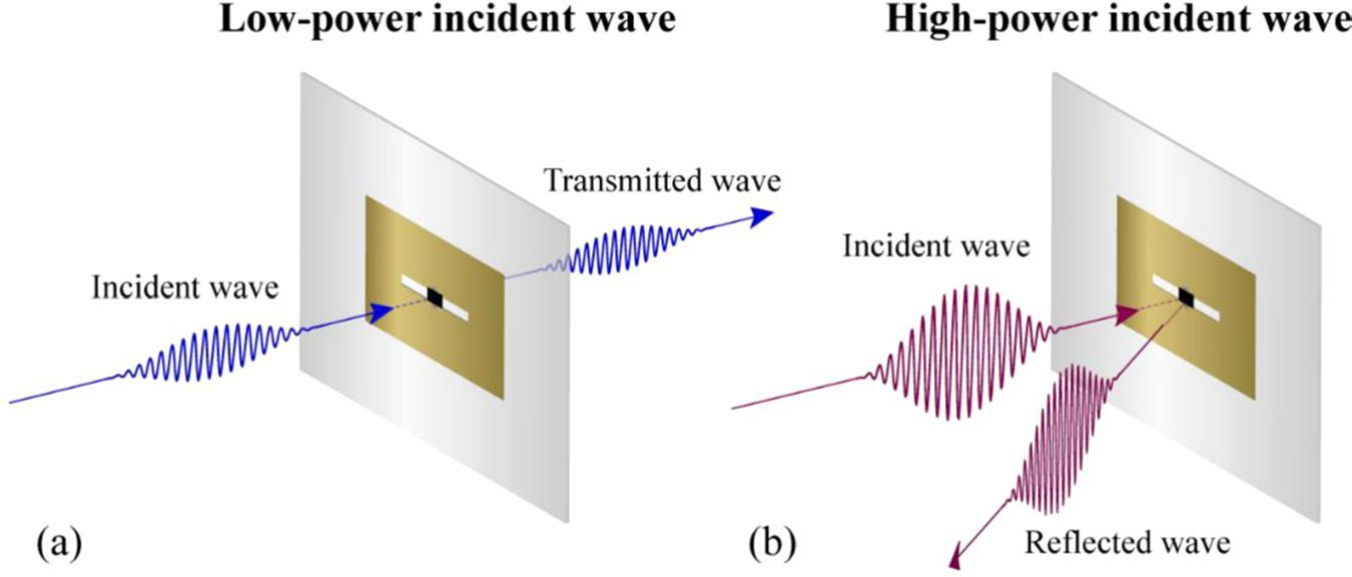
Working concept of the proposed RF-power-modulated active metamaterial: (**a**) transmission mode for a low-power incident wave and (**b**) reflection mode for a high-power incident wave.

In this study, a radio frequency (RF)-power-modulated active metamaterial was realized by loading a nonlinear Schottky diode onto a metamaterial. Its operating mode switches as a function of the incident power level by turning the Schottky diode on or off. The proposed metamaterial does not require any external DC power supply^22–27,33^. In contrast to the conventional design of nonlinear metamaterials^28–32^, the proposed structure is analogous to a two-dimensional (2D) planar surface, with the propagation direction being perpendicular to the surface. It is advantageous to construct a planar metasurface for free-space applications. The working concept of the proposed active metamaterial is depicted in Fig. 1. When a low-power RF wave is incident on the proposed metamaterial, the Schottky diode turns off. The metamaterial is then in the pass-band mode and passes 2 GHz signals, as shown in Fig. 1(a). When a high-power RF wave is incident, the diode turns on, and the proposed metamaterial reflects all frequencies since it is in the reflection mode (Fig. 1(b)).

## Results

### Design and simulation

**Figure 2:**
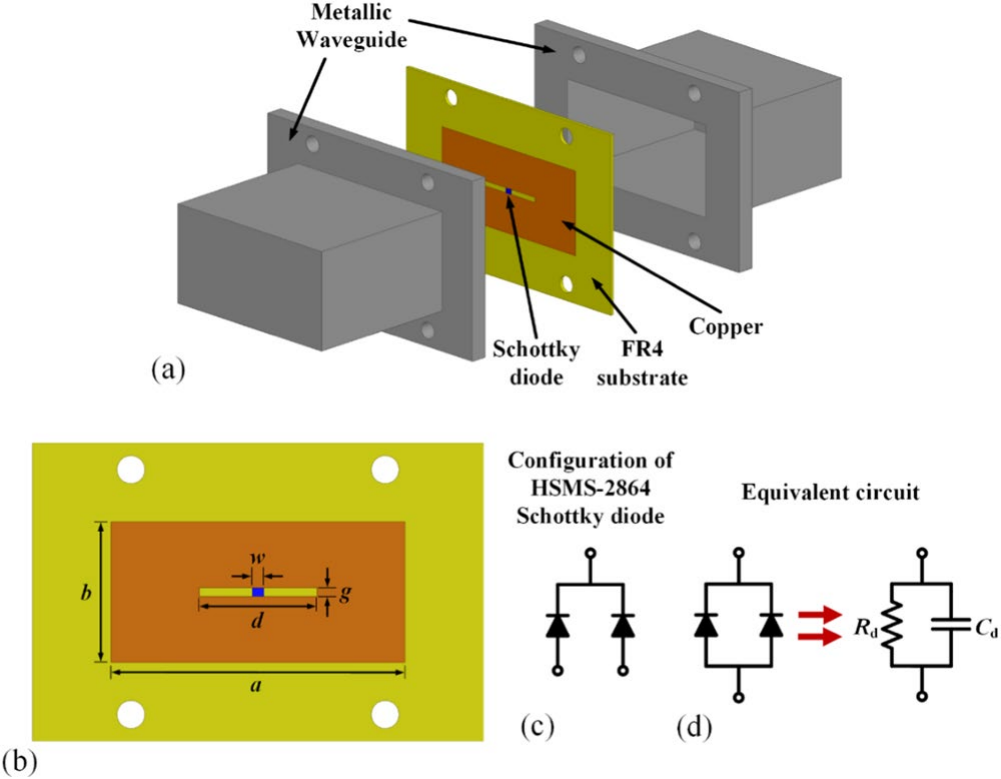
(**a**) Three-dimensional view of the proposed active metamaterial with two metallic waveguides. (**b**) Top view of the proposed metamaterial. The dimensions were *a* = 50 mm, *b* = 105 mm, *d* = 42 mm, *g* = 1.2 mm, and *w* = 1 mm, and the thickness of the FR4 substrate (*t*) was 0.8 mm. (**c**) Configuration of the HSMS-2864 Schottky diode. (**d**) Equivalent-circuit model. The circuit parameters were *C*_d_ = 0.56 pF and *R*_d_ = 100 KΩ and 1 Ω at low and high RF power levels.

The structure of the proposed RF-power-modulated active metamaterial was designed such that its responses could switch according to the power level of the incident wave. Figure 2(a) shows the proposed active metamaterial. As shown in Fig. 2(b), the metamaterial has a single horizontal metallic slot (σ = 5.8 × 10^7^ S/m) at the center of a 0.8 mm thick FR4 substrate (dielectric constant (*ε*_r_) = 4.4 and tangential loss (tan δ) = 0.02). For RF power modulation, a Schottky diode (Broadcom HSMS-2864) was embedded across the gap. This diode was a Schottky pair in common cathode configuration, as shown in Fig. 2(c). In this work, the two anodes of the Schottky diode were connected together. The diode was modelled as a parallel combination of a resistance and a capacitance (*R*_d_-*C*_d_)^34^, as shown in Fig. 2(d). The value of *R*_d_ changed with the incident power level. *R*_d_ changed from 100 KΩ to 1 Ω when the input power was varied from −30 dBm to +30 dBm, respectively. By contrast, *C*_d_ remained constant at 0.56 pF for all input power levels.

For this design, the Schottky diode was switched without a DC bias network, depending instead on the incident RF power. When an RF power was incident on the active metamaterial, it induced a surface current and generated a potential difference across the anode and cathode of the diode. The intensity of the surface current depended on the incident power level. Therefore, the response of the metamaterial depended on the power of the incident RF wave. In other words, it could be turned on or off by high or low incident RF power.

**Figure 3:**
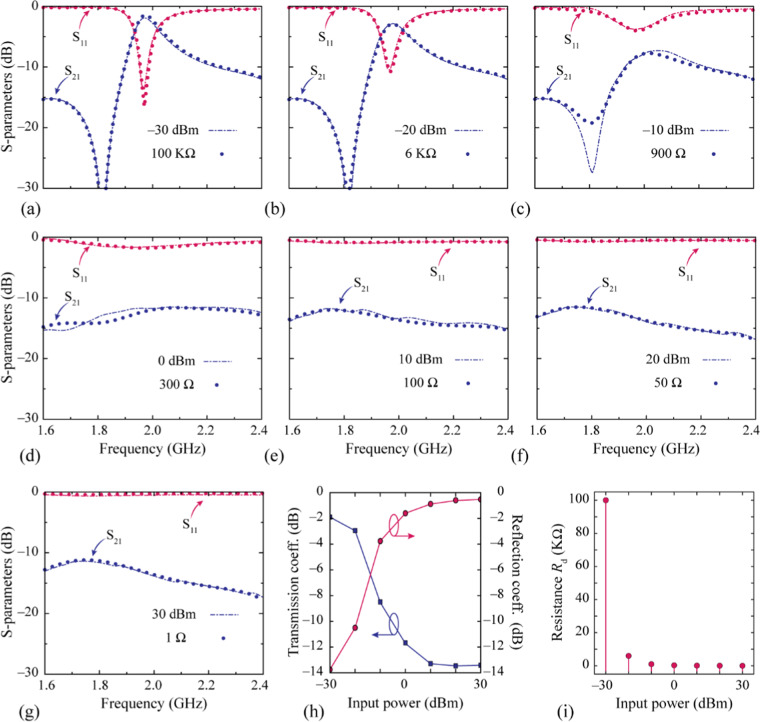
(**a**–**g**) Comparison of the simulated S-parameter results of the SPICE model at different power levels with those of the simplified RC circuit model at different resistance values. (**h**) Transmission and reflection coefficients at the resonant frequency for different input power levels. (**i**) Resistance values for different input power levels.

Because the full-wave simulation ANSYS High-Frequency Structure Simulator (HFSS) setup does not support a nonlinear SPICE model for the diode, we considered a simplified *RC* circuit for the Schottky diode pair. Since the value of the resistance depended on the externally applied bias current, we could change it as a function of the incident power level. The resistance value was determined from the S-parameter results by using the Keysight Advanced Design System (ADS). In Fig. 3(a–g), the S-parameters of the simplified *RC* circuit model for different resistances are compared with those of the SPICE model for different incident power levels from −30 dBm to +30 dBm. Figure 3(h) shows the transmission and reflection coefficients at the resonant frequency for different incident power levels. The response of the proposed metamaterial changed from the transmission mode to the reflection mode when the incident power increased from −30 dBm to +30 dBm. The equivalent resistance is shown as a function of the incident power level in Fig. 3(i). The S-parameter values of the two models are in good agreement, validating the proposed circuit model for other power levels.

**Figure 4:**
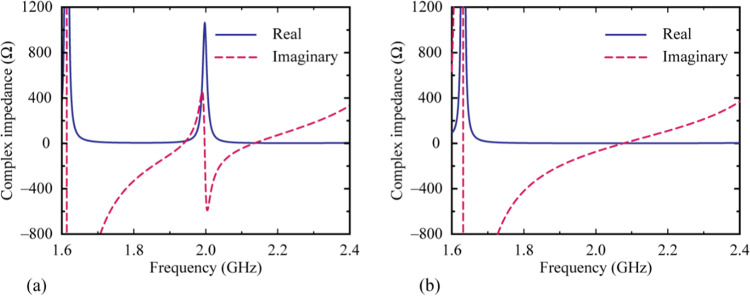
Complex impedance of the proposed RF-power-modulated metamaterial at different power levels: (**a**) low input power level (*R*_d_ = 100 KΩ) and (**b**) high input power level (*R*_d_ = 1 Ω).

A full-wave electromagnetic simulation was performed using the HFSS simulator. The Schottky diode was modelled as a lumped boundary, which represented as a parallel connection between the resistance and the capacitance (*R*_d_-*C*_d_). In the simulation, *R*_d_ was considered as 100 KΩ and 1 Ω for the low- and high-power modes, respectively. *C*_d_ remained constant at 0.56 pF for both modes. Figure 4 shows the complex impedance for both low and high input power levels. At the low input power level, the resonant frequency occurs near 2 GHz and results in a bandpass response since the impedance of the metamaterial matches that of the waveguide, as shown in Fig. 4a. At the high incident power level, there is no impedance matching, and the metamaterial operates in the reflection mode, as shown in Fig. 4b.

### Experimental verification

**Figure 5:**
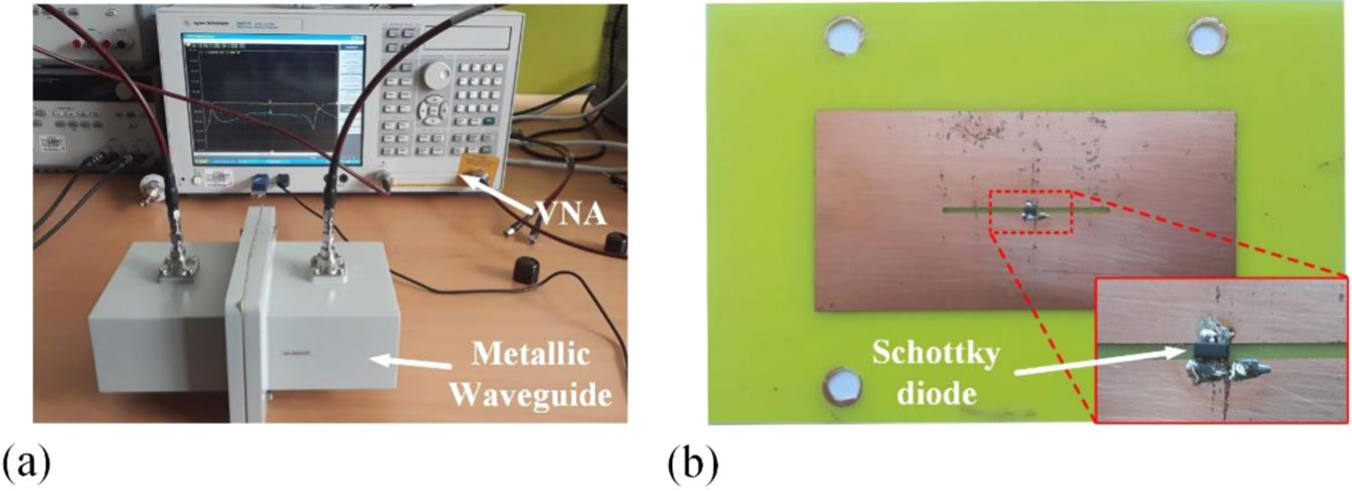
Photographs of the (**a**) S-parameter measurement setup and (**b**) fabricated sample.

**Figure 6:**
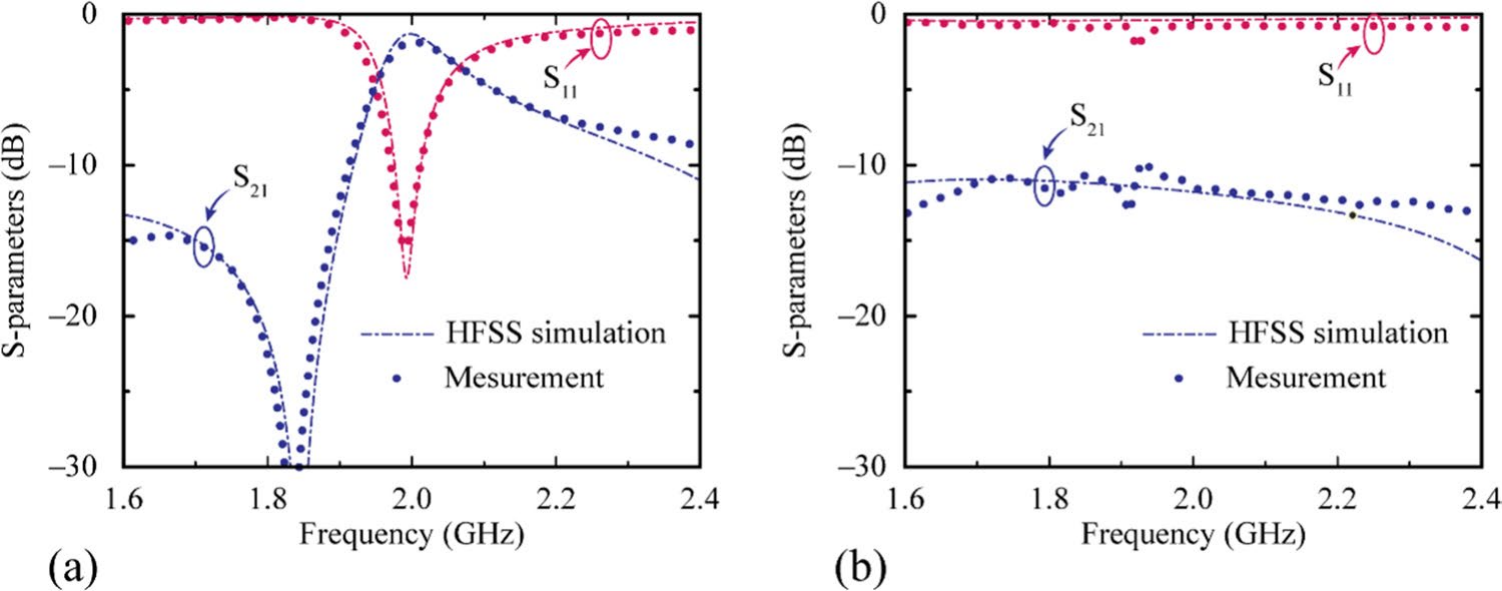
S-parameters of the proposed metamaterial obtained from the full-wave simulation and measurements for the (**a**) low-RF-power mode and (**b**) high-RF-power mode.

To validate the simulation results for the proposed metamaterial, a sample of the metamaterial was fabricated by using the printed-circuit-board etching process. Photographs of the measurement setup and fabricated sample are shown in Fig. 5. The Schottky diode was turned on when the RF power exceeded 0 dBm, and therefore, the S-parameters in the high-RF-power mode were measured by setting the input RF power to 10 dBm in the network analyzer. For the low-RF-power mode, the S-parameters were measured by setting the input RF power to −20 dBm. Figure 6 shows the S-parameter results obtained from the full-wave simulation and measurements. At the low RF power level, the proposed active metamaterial operated in the pass-band mode. At 2 GHz, the measured insertion and return losses were 1.83 and 15.55 dB, respectively. The simulated insertion and return losses were 1.32 and 15.02 dB, respectively. At the high RF power level, the proposed active metamaterial operated in the reflection mode. The measured insertion loss was 11.52 dB at 2 GHz, while the simulated insertion loss from the full-wave analysis was 12.13 dB. The ripples in the experimental results were due to the near-field coupling between the waveguide adapter and the metamaterial’s aperture. They can be avoided by using straight waveguides at each port. The response time of the proposed metamaterial depended on the Schottky diode, and the response time required to reach a steady-state response was approximately 3 ns. The experimental results demonstrated that the proposed active metamaterial was successfully modulated by the RF power level.

## Methods

### Simulation

The nonlinear analysis of the RF-power-modulated metamaterial was numerically performed by ANSYS HFSS and Agilent ADS. For instance, we first simulated the proposed structure in ANSYS HFSS. Wave ports were used to excite the incident wave at both ports of the rectangular waveguides and lumped port was inserted at terminals where the Schottky diode was supposed to be loaded. The S-parameter data from HFSS is imported to Agilent ADS and then full nonlinear analysis is performed with a nonlinear SPICE model of the Schottky diode.

The linear approximation analysis is carried out by replacing the diode with the proposed circuit model to determine the appropriate equivalent resistances for different input power levels. Full-wave electromagnetic results (Fig. 6) were obtained using ANSYS HFSS software with linear approximation values obtained from circuit solver.

### Measurements

Measurements for the fabricated sample were performed in a waveguide environment, and the sample was placed between two rectangular waveguides as shown in Fig. 5(a). The part number of the waveguides was 430WCAS, and they were procured from Chengdu AINFO Inc. Their frequency range was 1.7–2.6 GHz. The two rectangular waveguides were connected to an Agilent E5071C vector network analyzer (VNA). Since the proposed sample switched as a function of the incident power level, the S-parameters were measured by setting the input RF power to −20 and 10 dBm, which corresponded to the low and high RF power modes, respectively.

## Discussion

In summary, an RF-power-modulated active metamaterial is presented, and it was demonstrated by performing a full-wave electromagnetic simulation and using a circuit analysis. A nonlinear active component, a Schottky diode, was introduced on the metamaterial, and the dependence of the switching characteristics of the proposed metamaterial on the incident power level was determined. The proposed concept was investigated experimentally by fabricating a sample and testing it in a waveguide environment. A good agreement was obtained between the simulation and the measurement results. At the low incident power level, the EM wave could propagate through the structure, which passed a 2 GHz signal in the pass-band mode with an insertion loss of 1.83 dB. At the high incident power level, the proposed structure operated as a reflector, reflecting all frequencies of the incident wave. Since the proposed concept was demonstrated in the waveguide, but it can be extended to design a 2D metasurface by applying a periodic boundary. The EM responses of the periodic boundary might be slightly different from the waveguide results. However, the desired responses can be optimized by controlling the dimension of the metallic slot of a unit cell. In addition, this concept could be applied to the design of electromagnetic structures such as metasurfaces or metamaterial antennas whose responses should change as a function of the incident power level.
